# The use of partial exchange blood transfusion and anaesthesia in the management of sickle cell disease in a perioperative setting: two case reports

**DOI:** 10.1186/1752-1947-4-82

**Published:** 2010-03-05

**Authors:** Rhett Jaeckel, Matthias Thieme, Elke Czeslick, Armin Sablotzki

**Affiliations:** 1 Klinikum "St Georg" gGmbH, Department of Anaesthesiology and Intensive Care Medicine, Delitzscher Str, 04129 Leipzig, Germany

## Abstract

**Introduction:**

Homozygous sickle cell carriers have an increased perioperative mortality. Some indications may justify an exchange blood transfusion to reduce the proportion of haemoglobin S. The advantages of general blood transfusion in a perioperative setting have not been proven and thus remain controversial. It is not clear whether reducing the proportion of haemoglobin S minimizes perioperative complications or whether patients with sickle cell disease in a stable clinical condition benefit from an exchange blood transfusion in a perioperative setting.

**Case presentation:**

We report the case of two Angolan children aged 10 and 11 respectively, of African origin with sickle cell anaemia who underwent surgery to treat chronic necrosis, fistula of the bones and bone destruction. This presentation describes the perioperative course, including general anaesthesia. A partial exchange blood transfusion decreased S-haemoglobin levels from 81% to 21% and simultaneously treated the anaemia.

**Conclusion:**

There is a consensus that imbalances in homoeostasis, including operative procedures, can cause a critical exacerbation of sickle cell disease. The case presented here illustrates a strategy for successfully managing sickle cell disease in the perioperative period to minimize its complications. It is important for the anaesthesiologist to carefully manage pulmonary gas exchange and to ensure sufficient tissue perfusion, balanced fluid resuscitation and normothermia, while keeping in mind the level of organ impairment in order to prevent an acute exacerbation of sickle cell disease.

We performed a partial exchange blood transfusion due to the following factors: high haemoglobin S-fraction, anaemia, operating procedure at several sites, and difficult management of body temperature. Esmarch ischemia is an established tool for preventing uncontrolled blood loss. There is no known contraindication for this, but attention must be paid to prevent uncontrolled tissue ischemia and acidosis. The use of regional anaesthesia should be considered for postoperative pain management.

## Introduction

Disturbances in haemoglobin synthesis are some of the most common human hereditary disorders. There is an increased prevalence among the African and Asian populations, and more recently, immigration has led to an increase in the incidence of this disorder in Europe as well. In Germany, for example, there are currently an estimated 1000 patients with sickle cell disease (SCD) [1].

SCD is a haemoglobinopathy characterized by an abnormal haemoglobin variant termed haemoglobin S (HbS). HbS causes irreversible filamentous precipitation, which causes red blood cells to change shape, which in turn leads to circulation problems. Clinical symptoms include relapsing ischemic episodes, chronic haemolysis and a specific type of anaemia termed sickle cell anaemia. The cause of this hereditary disorder is a single amino acid substitution in the haemoglobin protein.

Heterozygous sickle cell carriers are relatively resistant to malaria, but homozygous patients are in danger of increased perioperative mortality and have a reduced life expectancy. In particular, conditions such as hypothermia, hypoxia, acidosis and dehydration in the perioperative period can cause an acute exacerbation of the disease [2,3]. Therefore, perioperative treatment must include measures to prevent these conditions, as well as measures to ensure safe general anaesthesia for these at-risk patients.

## Case presentation

We report the case of a 10-year-old Angolan boy with homozygous sickle cell anaemia. An international aid organization sponsored the medical treatment for the boy and his brother, who was one year older and who also had SCD. Both boys are of African origin.

The two boys were admitted to our hospital because of chronic necrosis at several sites and fistula of the long bones. On admission, the boys were severely ill and had been ravaged by the effects of chronic SCD. Their heights and weights were below the 3^rd ^percentile (compared with children of Central Europe), and they had muscle hypotrophy, signs of chronic hypoxia and chronic hepatitis B. The 10-year-old had malaria quartana, and his older brother had terminal renal insufficiency. Episodes of acute chest syndrome were not reported for both of them.

The 10-year-old boy was unable to walk because of multiple aseptic bone necrosis of the right tibia bone, the left femur and the lower leg. Imaging revealed pseudarthrosis, a fractured left fibula and a completely destroyed left tibia. An older healed fracture of his left femur was malpositioned. The lower leg bones were destroyed and could not be reconstructed, and an exarticulation had to be performed at the knee joint. In addition, multiple sequestrectomies had to be performed on the patient. Due to a hard estimation of blood loss and duration of the whole procedure, this kind of surgery is considered high risk.

On admission, the boy had the following laboratory parameters: severe anaemia, with 3.9 mmol/l haemoglobin and a haematocrit of 0.19; a 14.7% increase in reticulocytes; and positive results for the HbS solubility test. Haemoglobin high performance liquid chromatography (HPLC) revealed an HbS fraction of 81.7%, HbA_2 _of 4.7% and HbF of 3.0%. The C-reactive protein level was at 70.5 mg/litre. The overall bilirubin was 52 μmol/litre and the direct (conjugated) bilirubin was 21 μmol/litre. Hyponatraemia was 131 mmol/litre. The test for hepatitis B surface antigen was positive, as was the test for the hepatitis B envelope antigen. The result of the hepatitis B envelope antigen test was negative, but there was evidence of Plasmodium *malariae*. Sonography showed nodular parenchyma and cholecystitis. The boy weighed 22 kg.

During preoperative care, a partial exchange transfusion was performed because of the high percentage of HbS. The surgical risk was also valued as very high due to his reduced health condition and multiple foci with probable prolonged duration of surgery. Meanwhile, blood loss was hard to estimate. One day before surgery, 1200 ml of blood (equivalent to 70% of his blood volume) was removed via the femoral artery for over 200 minutes. The blood was replaced using red blood cell concentrate and normal saline (1:2) This partial exchange transfusion decreased the HbS fraction to 21.6%; afterwards, haemoglobin HPLC revealed that his HbA_2 _was 7.4% and HbF was 1.9%. The haemoglobin was 7.5 mmol/litre, and the hematocrit was 0.36.

On the day of the surgery, the boy received 3 mg of Midazolam intravenously. The oxygen saturation was monitored using pulse oximetry. His body temperature was monitored via a rectal temperature sensor and was kept stable within a tight range (37.0 ± 0.5°C) by increasing the temperature of the operating room, use of heat conduction with special equipment, and heat convection (warm touch).

General anaesthesia with endotracheal intubation was performed as total intravenous anaesthesia using Propofol and Fentanyl. Cisatracurium was used as a muscle relaxant. Surgery was performed with an arrest of the blood supply around the thigh. The overall blood loss during the procedure was approximately 600 ml and was substituted using a balanced electrolyte solution, hydroxyethyl starch, 1 transfusion unit of red blood cells and 1 transfusion unit of fresh frozen plasma. Circulation parameters were kept stable during the surgical procedure. Conditions that could have triggered an acute exacerbation of SCD, such as hypoxia, hypovolemia, hypothermia and hyperviscosity, were prevented by monitoring his vital signs and maintaining them within tight ranges. Anaesthesia from the beginning up to the end of surgery lasted 80 minutes.

Postoperative care, including fluid management and weaning off the respirator with extubation on the day of the surgery, was provided by the hospital's Pediatric Intermediate Care Unit. Pain was managed using Paracetamol and Piritramid intravenously as needed.

After the surgery, the boy complained of a relapsing upper abdominal pain. Laboratory parameters showed increased markers for cholestasis. After an endoscopic retrograde cholangiopancreatography, a cholecystectomy was performed four weeks after the initial orthopaedic surgery, which revealed severe cholecystitis. Without further transfusions at the time of abdominal surgery, the HbS fraction was 34.5%; HPLC revealed that HbA_2 _was 1.1% and HbF was 1.5%. The haemoglobin was 7.1 mmol/litre, and the hematocrit was 0.35. Due to the modest level of Hbs fraction, we planned a substitution of blood loss. During cholecystectomy, which lasted for 45 minutes, one unit of red blood cell was transfused. Table 1 presents the time course of haematological data and surgical procedures.

**Table 1:** Patient 1: Time course of haematological data and procedures.

Day	Hb [mmol/litre]	Hk [%]	HbS [%]	Measure
Admission	3.5	18	81.7	

day 2	4.0	20		

day 8	7.5	36		Exchange transfusion

day 9 pre-surgery	6.4	30	21.6	Orthopaedic surgery

day 9 post-surgery	7.1	34		Transfusion of 1 unit red blood cell

day 13	6.3	32		

day 16	7.1	33		

Day 22	6.6	31		

Day 28	6.6	31		

Day 31	5.9	28		

day 38 pre-surgery	6.0	28	34.2	Cholecystectomy

day 38 post-surgery	6.7	31		transfusion of 1 unit red blood cell

day 56 discharge	5.6	27		

Meanwhile, the boy's 11-year-old brother was suffering from heterogeneous sickle cell disease with a lower HbS fraction. On admission the boy had the following haematological parameters: severe anaemia at only 2.0 mmol/litre haemoglobin and a haematocrit of 0.09. HPLC revealed an HbS fraction of 32.3%, HbA_2 _of 2.3%, and HbF of 1.1%. He had to undergo several surgeries, including sequestrectomies, drilling of the medullary cavity and implantation of special devices to release antibiotics. Due to HbS fraction of 32.3%, we performed a substitution rather than exchange of the blood he lost. In the course of surgeries he lost a lot of blood, on the order of his whole blood volume, which was substituted successfully with red blood cells, fresh frozen plasma and balanced electrolyte solution. Table 2 shows the time course and haematological data for this boy.

**Table 2:** Patient 2: Time course of haematological data and procedures

Hb [mmol/l]	HK [%]	HbS [%]	Measure
2.0	0.09	32.3	Transfusion of 3 units of red blood cells

4.8	0.25		

5.3	0.24		Transfusion of 2 units of red blood cells

5.0	0.24		Start dialysis, transfusion of 2 units of red blood cells

6.9	0.31		

5.6	0.27		Sequestrectomy tibia, transfusion of 2 units of red blood cells

5.5	0.26		

4.6	0.22		Sequestrectomy femur, transfusion of 1 unit of red blood cell

4.9	0.22		Transfusion of 2 units of red blood cells

6.6	0.31		Sequestrectomy humerus, transfusion of 1 unit of red blood cell

5.6	0.27		

5.0	0.23		Debridement tibia, transfusion of 2 units of red blood cells

5.4	0.26		

4.5	0.23		Debridement humerus, transfusion of 1 unit of red blood cell

3.7	0.18		Transfusion of 2 units of red blood cells

5.3	0.25		Debridement humerus, transfusion of 1 unit of red blood cell

4.4	0.21		General anaesthesia to insertion of central venous line

3.9	0.18		Transfusion of 1 unit of red blood cell

5.2	0.24		

On admission he suffered from severe renal insufficiency and had to be treated with dialysis. The boy underwent a total of seven surgical procedures and did not develop any complications known to be associated with SCD. The surgeries lasted between 30 and 90 minutes. General anaesthesia was performed on the patients as described previously.

## Discussion

SCD is a hereditary haemoglobinopathy characterized by a mutation in the β-globulin gene on chromosome 11. This mutation leads to the synthesis of HbS, in which the hydrophobic valine at position 6 of the 146-amino acid haemoglobin protein is replaced by the hydrophilic amino acid glutamine. This replacement changes the structure of haemoglobin: It becomes destabilized and tends to precipitate when deoxygenated. This in turn causes erythrocytes to take on the typical sickle cell shape and also increases membrane fragility. Dehydration due to an increased intracellular haemoglobin concentration increases HbS polymerization. In homozygous sickle cell carriers, the HbS ranges from 75% to 95%. Such carriers have increased perioperative mortality and a decreased life expectancy for the patient. About 30% suffer from a rapid course. Heterozygous sickle cell carriers produce both HbS and normal HbA and are usually asymptomatic [1]. Because of their resistance to Plasmodium falciparum, heterozygous carriers are more resistant to malaria and have a selective advantage in places where the disease is rampant [2].

Despite their uniform genotype, heterogeneous carriers have highly variable phenotypes. Relapsing capillary obstruction causes ischemic damage in many organs, which leads to aseptic bone sequestration, chronic osteomyelitis, renal insufficiency and fibrotic transformation of the spleen. This damage is accompanied by loss of function, decreased resistance to infection, cutaneous ulceration, retinopathy, acute cerebral and cardial circulatory disorders, fibrotic lung transformation and pulmonary hypertension. Acute life threatening episodes occur when the carrier is affected by haemolytic and pain crises, splenic sequestration, lung problems (acute thoracic syndrome) and corpus cavernosum (priapismus). Because the erythrocytes affected by SCD have shorter life cycles (< 21 days), carriers suffer from chronic haemolysis accompanied by anaemia, hyperbilirubinemia and cholecystolithiasis. In fact, the most common surgical procedures in patients suffering from SCD are cholecystectomies and orthopaedic surgery [3,4].

In addition to classical triggers, the activation of endothelial factors, immunological responses and other factors modulate the onset and course of the disease and influence its pathophysiology. Nevertheless, there is a consensus that imbalances in homoeostasis can cause critical exacerbation of SCD. For that reason, it is essential to maintain normovolemia, normothermia and normoxemia during anaesthesia and the perioperative period [5,6].

The clinical symptoms of SCD, which start in early childhood, are splenomegaly, haemolytic anaemia and relapsing pain. A diagnosis of SCD can be confirmed using electrophoresis or chromatography [7]. Before elective surgery, it is important to quantify SCD-related parameters and determine whether organ insufficiencies exist. Parenteral substitution with balanced electrolyte solutions before surgery prevents dehydration and the use of balanced volume substitution prior to surgery and until ingestion is possible can avoid asymptomatic fluid deficiencies. In cases of longer procedures with increased fluid shifts, a urinary catheter is indicated to monitor urinary volume. Hypoxemia due to hypoventilation must be avoided by way of adequate monitoring throughout the perioperative period, and benzodiazepines should be given to reduce stress. Sufficient denitrogenization minimizes the risk of hypoxemia during the induction of anaesthesia. There is no evidence that alkalinization or intraoperative hyperoxygenation with prolonged oxygen administration after surgery provides any benefit.

Just as hypothermia can be harmful because of increased viscosity, vasoconstriction and the resulting increased oxygen consumption and hyperthermia can escalate precipitation, thus jeopardizing the patient's condition. Therefore, the patient's temperature must be monitored constantly and measures must be taken to prevent heat loss. In addition to basic haemodynamic monitoring, pulmonary gas exchange must also be continuously monitored by spectrometric pulse oximetry and capnometry. Depending on the clinical situation, an artery catheter can be used to analyze blood gas and monitor the haemoglobin level during the surgical procedure.

Because patients with SCD generally have a history of chronic pain and thus a history of using analgesics, some may have a tolerance to opioids. When anaesthesia is no longer needed, optimal fluid balance, analgesia, normothermia and sufficient spontaneous breathing activity are essential preconditions both for extubation and to prevent dangerous shivering during postoperative care. Postoperative care should ideally be given in an intermediate or intensive care unit.

For individuals with SCD, Repeated blood transfusions can reduce the frequency of ischemic complications (especially strokes) and adolescence retardation [8]. The degree to which HbS is reduced seems to generally correlate with reduced complication rates [9]. Based on pathophysiology and clinical experience, the substitution of oxygen carriers and reduction of HbS below 30% to 40% is recommended [6,10]. The advantages of blood transfusions have not been proven and thus remain controversial [5]. Koshy *et al*. described a statistically significant reduction of SCD-dependent complications from 12.9% to 4.8% using blood transfusions [4], and Neumayr *et al*. reported that transfusions decreased complications from 18% to 9% [11]. In contrast, Vichinsky *et al*. [3] compared aggressive exchange blood transfusion (which reduced HbS to < 30%) to a conservative transfusion regime (which increased haemoglobin to 6.3 mmol/litre) and found out that the complication rate (which was about 15%) did not differ. However, transfusion-associated complications in patients who receive aggressive exchange blood transfusions were 50% higher than in patients receiving conservative transfusions. An analysis of patients undergoing cholecystectomies and orthopaedic procedures indicated that an aggressive transfusion regime is not always advantageous [12]. Thus, the physician must consider whether transfusion associated complications, especially alloimmunization and increased ferrous load, might increase the perioperative risks to an unacceptable level [3].

It is not clear whether reducing the proportion of HbS minimizes perioperative complications, or whether patients with SCD in a stable clinical condition benefit from an exchange blood transfusion in the perioperative setting. In particular, there is no positive indication that exchange blood transfusion in patients undergoing minor surgery and who have adapted rather well to chronic anaemia can minimize complications. Our second case demonstrates this fact impressively. SCD associated complications were successfully avoided by the substitution of perioperative blood loss with red blood cells. Some indications, such as cerebral infarction, occlusion of the mesenteric arteries and tolerance to analgesia before a major surgery may justify an exchange blood transfusion to reduce the proportion of HbS to below 30% [1,6,10].

Reports in the literature do not show that one anaesthesia technique is better than others. Koshy *et al*. reported an increased incidence of complications using regional anaesthesia, but this was likely due to the high-risk obstetric patient population [4]. Other researchers did not find an increased risk with regional anaesthesia when performed according to the same safety standards used for general anaesthesia [13].

Esmarch ischemia is an established tool for preventing uncontrolled blood loss and was used in the orthopaedic surgery described in this case report. There is no known contraindication for this, but attention must be paid to prevent uncontrolled tissue ischemia and acidosis [14].

## Conclusions

SCD is a common hereditary affliction that is more widespread among peoples in sub-Saharan Africa. Increased population migration has increased the prevalence of this disease in other countries as well. SCD causes progressive damage to multiple organs. Although the concept of classical SCD triggers is less important today, the effect of the disease on the carrier's vascular system determines the aetiology of the disease.

Patients are at risk in the perioperative period because the physiological environment is disrupted. Thus, in order to prevent an acute exacerbation of SCD, the anaesthesiologist should carefully manage pulmonary gas exchange, ensure sufficient tissue perfusion, balanced fluid resuscitation and normothermia, and keep in mind the level of organ impairment. The use of regional anaesthesia should be considered for postoperative pain management, and preoperative transfusions can reduce ischemic complications during chronic treatment.

In this case we performed a partial exchange blood transfusion due to high HbS fraction, operating procedure at several sites, and a body temperature that was difficult to manage. Intraoperative blood transfusion should be used to compensate for blood loss and reduce anaemia. The case presented here illustrates a strategy for successfully managing SCD in the perioperative period that would minimize complications.

## Abbreviations

HbS: haemoglobin S; HPLC: high performance liquid chromatography; SCD: sickle cell disease.

## Consent

Written informed consent was obtained from the patients' parents for publication of this case report and any accompanying images. A copy of the written consent is available for review by the Editor-in-Chief of this journal.

## Competing interests

The authors declare that they have no competing interests.

## Authors' contributions

RJ was involved in conceiving, designing and writing this manuscript. MT was involved in providing a description of the perioperative procedures. EC was involved in writing the introduction, analysis and data interpretation sections of this manuscript. AS provided the critical revisions and final corrections for this manuscript. All authors have read and approved the final manuscript.
